# What Are the Clinical Implications of the SARS-CoV-2 Variants: 5 Things Every Cardiologist Should Know

**DOI:** 10.1016/j.jacbts.2021.02.009

**Published:** 2021-02-26

**Authors:** Adriana M. Rauseo, Jane A. O’Halloran

**Affiliations:** Division of Infectious Diseases, Department of Medicine, Washington University School of Medicine, St. Louis, Missouri, USA

**Keywords:** coronaviruses, SARS-CoV-2, variants

## Abstract

New variants of the severe acute respiratory distress syndrome coronavirus 2 (SARS-CoV-2) are emerging around the world and causing widespread concern regarding their ability to escape natural or vaccine-induced immunity and available therapeutics. Here, we will briefly review the potential clinical implications of these new variants.

Since its emergence in late 2019, severe acute respiratory syndrome coronavirus 2 (SARS-CoV-2) has given rise to a global pandemic that remains uncontrolled, in part related to the virus’ ability to adapt. Through genomic surveillance of SARS-CoV-2, a number of variants have recently been identified. Although the emergence of viral variants is anticipated with ribonucleic acid (RNA) viruses such as SARS-CoV-2, it is critically important to determine the impact of these variants on pandemic control and, in particular, on therapeutics and vaccines. This primer is not intended as an exhaustive review of all SARS-CoV-2 variants. Instead, here, we use some of the known variants to illustrate the potential challenges arising from their emergence.

## What are Viral Variants and Why are They Important?

RNA viruses, including coronaviruses, acquire mutations quickly during viral replication in the host cytoplasm. Despite the presence of RNA proofreading activity that yields high replication fidelity, coronaviruses have several features that provide opportunities to acquire mutations compared with DNA viruses (1). The term mutation refers to the change in amino acid sequence of the virus. For example, one of the first described mutations of SARS-CoV-2 was an aspartic acid-to-glycine substitution at amino acid position 614 of the spike (S) protein (referred to as D614G). Viral genomes that differ in sequence are referred to as variants. New variants can potentially lead to challenges in clinical management due to greater disease transmissibility and virulence, as well the ability to cause disease in subjects who received vaccines that were developed for the wild-type SARS-CoV-2 (1).

## What are the Main SARS-COV-2 Variants and How Were They Identified?

Since it was first identified in Wuhan, China, the SARS-CoV-2 genome has developed several mutations. Of particular clinical relevance are those mutations in the S protein. The S protein is responsible for binding the virus and is the target for neutralizing antibodies during infection as well as antibody-containing treatments and vaccines. Neutralizing antibodies bind to the S protein through the receptor-binding domain (RBD), and by doing so, these antibodies prevent the virus from attaching to the angiotensin-converting enzyme 2 (ACE2) receptor on human cells (2).

Early in the pandemic, the S protein mutation outside of the RBD (D614G) emerged and became the dominant circulating variant globally by June 2020 (1). Several variants have since been identified, with some designated “variants of concern” due to their potential clinical significance (Table 1). Although a consistent nomenclature for the variants has not been established, the most recognized nomenclature focuses on viral phylogenetic lineages, and 3 variants have recently been highlighted thus far, including B.1.1.7, B.1.351, and P.1 (1). They all share the D614G mutation in addition to other novel mutations of the S protein, including 2 other mutations in the RBD that are of particular concern. One of these mutations, N501Y, increases affinity for ACE2 receptor (i.e., makes it stickier) (2), and E484K is considered an escape mutation, as it potentially reduces antibody neutralization sensitivity, thereby evading the immune system (3).

**Table 1:** Comparison of the 3 Main SARS-CoV-2 Variants

Lineage Classification	First Identification	Key Mutations	Geographic Distribution	Potential Clinical Implications
B.1.1.7	September 2020 in the United Kingdom	• 69/70 deletion • 144Y deletion • N501Y • A570D • P681H • D614G • E484K	62 countries in Europe, Asia, and the United States	• Up to 50% increased transmissibility • Potential increased virulence • Low concern for decreased vaccine efficacy
B.1.351	December 2020 in South Africa	• K417N • E484K • N501Y • D614G	Africa, Europe, Asia, Australia, and the United States	• Increased transmissibility • No evidence of increased virulence • Potential for immune escape after natural infection and small effect on potency of vaccine-induced antibodies
P.1	January 2021 in travelers from Brazil at a Japanese airport	• E484K • K417N/T • N501Y • D614G	Brazil’s Amazonas, the Faroe Islands, South Korea, and the United States	• Unknown effect on transmissibility or virulence • Unknown potential for immune evasion

SARS-CoV-2 = severe acute respiratory syndrome coronavirus 2.

Despite sharing some of the same mutations, the 3 main variants emerged independently of each other. In September 2020, the B.1.1.7 variant was first detected in the United Kingdom and has rapidly spread around the world (3). The multiple mutations (17 in total, 8 in the S protein) of this variant suggests that it evolved over a prolonged period, likely in a chronically infected host (1). The B.1.351 variant was detected in December 2020 in South Africa and the P.1 variant was first identified in travelers from Brazil in January 2021 during routine airport screening in Japan. Both variants share several mutations with B.1.1.7, including E484K and N501Y (Table 1) (3).

## Do SARS-COV-2 Variants Affect Transmissibility and Virulence?

The B.1.1.7 variant has different biological properties compared with previous variants. It has accounted for approximately 28% of SARS-CoV-2 cases in England, with population genetic models suggesting that it spreads 56% more quickly than other variants (1). The B.1.351 and P.1 variants have also been associated with increased transmissibility, and all 3 variants have now been detected in the United States (3). Heightened transmissibility is believed to be related to the D614G mutation, as it enhances binding to ACE2 receptor, increasing probability of infection, replication with higher viral loads in the upper airways, and transmissibility when compared with wild-type (1). As noted, the N501 mutation is of particular concern because it also potentially increases affinity for ACE2, which also means that it is more transmissible (1,2).

Furthermore, the P.1 variant has been linked to reinfection. It was detected in 42% of cases during a devastating surge in Manaus, Brazil, where it was estimated that 76% of the population had already been infected with SARS-CoV-2 (3). These reinfection cases might be the consequence of a limited and transitory protective immunity induced by the index infection, or of more concern, they might reflect the variant’s ability to evade the previous immune responses, presumably due to E848K mutation.

The possibility of worse outcomes due to these variants is currently being studied carefully. At the time of this writing, the data are inconclusive. Preliminary studies disclosed that the B.1.1.7 strain was not associated with increased hospitalization, 28-day case fatality, or reinfection (4); however, more recent data suggest increased mortality compared with other variants (3). At this time, there is no evidence to suggest that B.1.351 has an impact on disease severity, and there are no data available for the P.1 strain (3).

## Will These Variants Impact the Performance of Diagnostic Tests?

Another potential consequence of emerging variants is their ability to evade detection by diagnostic tests. Most available reverse-transcription polymerase chain reaction– based tests have been designed to detect specific RNA sequences found in the viral genome. These molecular tests are highly sensitive, as they identify multiple SARS-CoV-2 genetic targets, making them less vulnerable to potential pitfalls. However, no test is perfect. False negative results are possible if mutations occur in the parts of the virus’ genome assessed by that specific test. For example, a mutation present in the B.1.1.7 variant (69/70 deletion) has been found to affect the performance of some diagnostic molecular assays (4). Reporting any decrease in assay performance will be critical. In cases with high clinical suspicion and negative test result, repeat testing with a different test could be considered.

## Are There Any Implications for Therapeutics and Vaccine Efficacy?

One of the greatest concerns with emerging SARS-CoV-2 variants is the potential to escape natural or vaccine-induced immunity. A number of the S protein mutations have raised concern for escape from neutralizing antibodies produced by convalescent sera or monoclonal antibodies and potential decrease in protection by vaccines. This may also mean that patients with a prior SARS-CoV-2 infection could become reinfected with emerging new variants. The effectiveness of other existing therapies such as remdesivir should not be altered, as the target for this drug has not been associated to the known mutations.

In vitro studies have shown that neutralizing antibodies in convalescent sera have a lack of potency against the B.1.351 variant. Cocktails of monoclonal antibodies binding different areas in the S protein appear to retain activity against some of these emerging variants, although they may be less effective against B.1.351 (2). With respect to coverage of new variants by the current approved messenger RNA (mRNA) vaccines, a recent study showed that there was effective neutralization activity with the Pfizer-BioNTech vaccine (BNT162b2) in the presence of N501 mutation, which includes the B.1.1.7 variant. However, the efficacy of this vaccine substantially decreases in the presence of the E484K mutation (5). This effect was also seen in sera from volunteers who received the Moderna (mRNA-1273) vaccine (2). Additional in vitro studies have shown that there is reduced neutralizing activity against B.1.351 in the sera of subjects who received any of the mRNA vaccines (5). It is important to note that these in vitro experiments do not necessarily provide the full picture when it comes to natural or vaccine-acquired immunity, as there are many other components of immune memory that could prevent reinfections, including T cell responses. At the time of writing, data are not available on the impact of emerging variants on the adenovirus vector vaccines or the purified protein vaccines. However, as these also target the S protein, similar concerns exist, particularly highlighted by the recent reports of variable efficacy across regions in phase 3 trials of the Janssen (Ad26.COV2-S) and Novavax (NVX-CoV2373) vaccines.

In the current primer, we have described the main emerging variants of SARS-CoV-2 and the potential impact on existing therapies. It is expected that SARS-CoV-2 will continue to acquire new mutations that will give rise to new variants. For the present, there is cause for optimism with respect to the efficacy of the current generation of vaccines against some of the emerging variants of SARS-CoV-2. However, it should be emphasized that we do not have detailed knowledge about the efficacy of the vaccines with many of the emerging variants. Given that each newly infected person represents an opportunity to acquire new mutations and develop novel variants, slowing the spread of the virus through continued public health interventions not only is critical to prevent further morbidity and mortality, but also can potentially impact the emergence of viral variants.

## Funding Support and Author Disclosures

Both authors have reported that they have no relationships relevant to the contents of this paper to disclose.
